# Electronic and Optical Sensitivity of Aluminum-Doped Boron Phosphide Monolayers for DNA Base Detection: A First-Principles Study

**DOI:** 10.1021/acsomega.5c08476

**Published:** 2025-12-02

**Authors:** Sin Ye, Chen-Hao Yeh

**Affiliations:** Department of Materials Science and Engineering, 34902 Feng Chia University, No. 100, Wenhwa Rd. Seatwen, Taichung 407102, Taiwan

## Abstract

Genetic testing has become increasingly important for the early diagnosis of diseases, as it can significantly improve treatment success rates and patient survival. A new type of two-dimensional (2D) material, the boron phosphide (BP) monolayer, has recently been recognized as a semiconductor material with excellent electronic properties, high surface reactivity, and environmental friendliness. In this study, density functional theory (DFT) calculations are employed to investigate the adsorption behavior of nucleobases (A = Adenine, T = Thymine, C = Cytosine, G = Guanine) on an aluminum-doped BP monolayer (Al-doped BP). The optical sensitivity, electronic sensitivity, and work function of the material are also analyzed. The results demonstrate that the presence of aluminum enhances the adsorption ability of the BP monolayer, especially in the case of the adsorption of Adenine, which presents the highest adsorption energy of −2.27 eV. Additionally, the adsorption of Adenine on Al-doped BP shows remarkable optical sensitivity with significant changes, and both Adenine and Guanine exhibit the largest conductivity change on the Al-doped BP monolayer. These computational results contribute to a better understanding of the application mechanisms of aluminum-doped BP materials in detecting nucleobases. The findings indicate that the Al-doped BP monolayer demonstrates superior selectivity toward Adenine, making it a promising candidate for biosensor applications.

## Graphical abstract

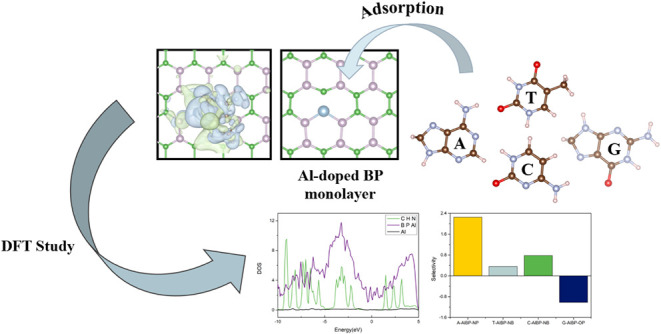

## 1 Introduction

Over the past decades, despite significant advances in medical technology, changes in modern lifestyle, such as increased consumption of processed foods, exposure to environmental pollutants, and prolonged chronic stress, have led to cellular damage and genomic instability.^1–4^ These long-term but often overlooked factors have contributed to a steady rise in the number of cancer- and gene-related diseases. Therefore, there is an urgent need for practical detection tools that can identify early molecular changes and help prevent disease development.^5,6^

Recent studies have highlighted that two-dimensional (2D) materials have gained extensive attention owing to their exceptional electrical properties, large surface-to-volume ratios, and inherent structural characteristics, making them highly suitable for applications in the sensitive and rapid detection of biomolecules.^7^ In particular, graphene, transition metal dichalcogenides (TMDs), hexagonal boron nitride (h-BN), MXenes, and boron phosphide (BP) are among the most widely used 2D materials in sensor applications.^8–11^ Although their properties are promising, many of these 2D materials still face limitations, such as weak gas-molecule interactions, low selectivity, or the requirement of complex surface modifications to enhance sensitivity.^12–14^

In literature, numerous studies have explored the sensing behavior of DNA nucleobases, including Adenine (A), Thymine (T), Cytosine (C), and Guanine (G), using various one-dimensional and two-dimensional nanomaterials.^15–20^ For example, Roondhe and Jha reported that a planar haeckelite BN sheet exhibits promising sensitivity toward Guanine, demonstrating its potential as a selective biosensor.^20^ Similarly, Kumar et al. investigated the adsorption of nucleobases on delta tellurene monolayers, showing that physisorption governs the sensing mechanism.^21^ To improve chemisorption and enhance detection sensitivity, Cruz-Irisson and coworkers examined the selective sensing of DNA/RNA nucleobases on metal-functionalized silicon nanowires, revealing that transition-metal decoration significantly increases interaction strength and selectivity, particularly for Adenine and Guanine.^16^ These studies collectively highlight the importance of material dimensionality and surface functionalization in tuning the adsorption characteristics and electronic responses of nanoscale biosensors designed for the efficient detection of DNA bases.

Accordingly, in this study, newly proposed 2D materials, Al-doping BP monolayers, have emerged as promising candidates due to their ability to significantly enhance the adsorption energy, optical sensitivity, and electrical sensitivity of pristine BP.^22,23^ Specifically, aluminum doping can effectively modulate the band gap and increase the carrier concentration, thereby enhancing the electrical conductivity and sensitivity essential for sensing applications.^24–27^ Moreover, the incorporation of Al atoms introduces additional active sites on the material’s surface, which strengthens molecular adsorption interactions-particularly with nucleobases. These modifications collectively improve charge transfer efficiency and significantly enhance the selectivity and responsiveness of the material, positioning Al-doped boron phosphide (Al-doped BP) as a promising candidate for gas sensing applications.^28–31^

To assess the sensing capabilities of Al-doped BP monolayers, four nucleobases-Adenine, Thymine, Cytosine, and Guanine-were selected as target analytes.^32–34^ These molecules are not only biologically significant as the fundamental constituents of DNA and RNA, but they also feature diverse functional groups capable of interacting with sensing surfaces.^35,36^ Our analysis included the calculation of the adsorption energies and an in-depth examination of the electronic properties of these gas molecules on the BP monolayer.^37–42^ This examination includes adsorption energies, electronic band structures, the projected density of states (PDOS), and electron density difference (EDD) calculations. Furthermore, to evaluate the optical sensitivity of the BP monolayer, we analyzed changes in its optical absorption characteristics before and after gas adsorption, which reveal how molecular interactions affect the material’s optical response.^43–46^

## 2 Results and Discussion

### 2.1 Optimized Adsorption Structures of the Nucleobase Molecules on Pristine BP and Al-doped BP Monolayers

The unit cell of boron phosphide (BP) is shown in Figure S1a, in which we built an orthorhombic primitive cell with the *Pmm2* space group. The boron phosphide unit cell contains two boron and two phosphorus atoms, and the calculated lattice parameters are *a* = 3.22 Å and *b* = 5.57 Å, having a similar trend to the previously reported result.^47^ Additionally, we calculated the elastic constants of boron phosphide, and the calculated values for C11, C22, C12, and C66 are 166, 146, 26, and 60 N/m, respectively. The elastic constants reach the mechanical stability criteria, and the calculated Young’s modulus and Poisson’s ratio are 141 N/m and 0.21, respectively. The mechanical properties of boron phosphide also exhibit a similar trend to the previously reported results.^47^ To model the boron phosphide monolayer, a (4 × 2) supercell (*a* = 12.88 Å and *b* = 11.14 Å) was constructed with a vacuum spacing of 15 Å along the *z*-direction to minimize spurious periodic interactions, as shown in Figure S1b.

Figure 1a,b depicts the top views of the pristine BP supercell and Al-doped BP monolayer supercell structures, respectively. Doping with an Al atom can effectively improve the BP monolayer’s adsorption performance. Boron was selected as the substitution site for doping with a single Al atom due to the same valence electrons. We further calculated the adsorption energies of Adenine (A), Thymine (T), Cytosine (C), and Guanine (G) on both pristine BP and Al-doped BP systems. The structures of the A, T, C, and G nucleobases are shown in Figure 2.

**Figure 1:**
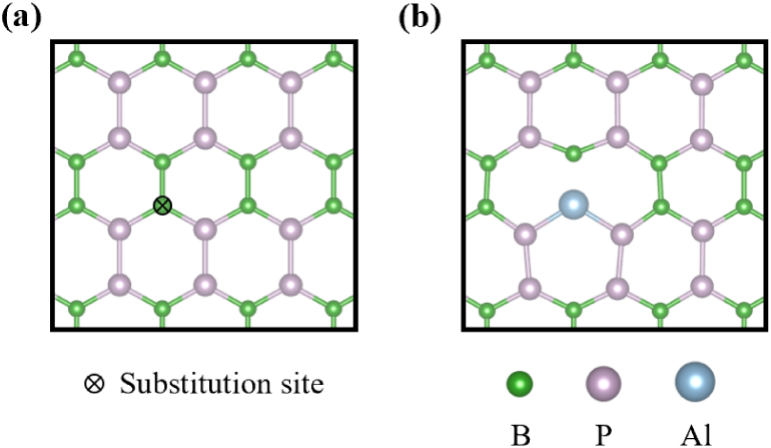
Top views of (a) pristine and (b) aluminum-doped BP monolayers.

**Figure 2:**
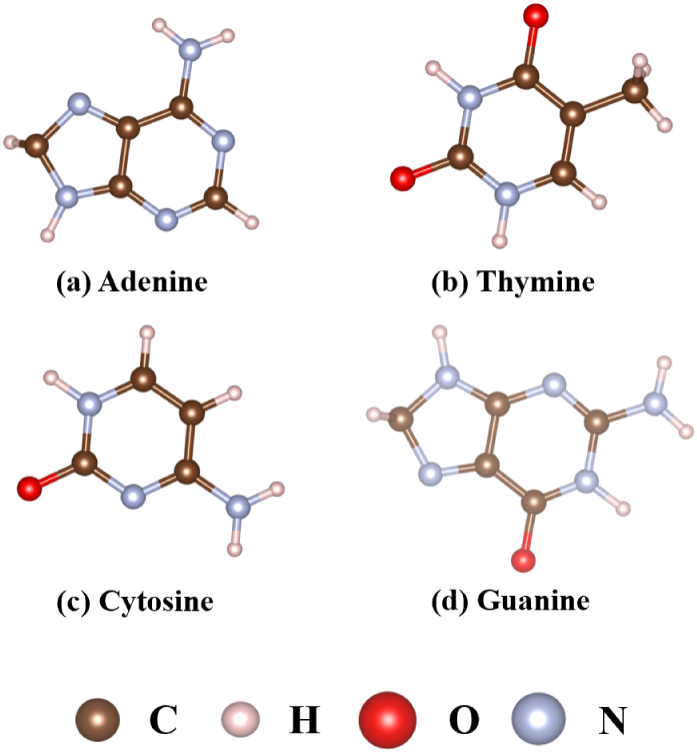
Optimized nucleobase structures: (a) Adenine, (b) Thymine, (c) Cytosine, and (d) Guanine.

The calculated C–H, N–H, C–C, C–N, and C–O bonds in all the DNA base molecules are about 1.09–1.10 Å, 1.01–1.02 Å, 1.36–1.50 Å, 1.32–1.39 Å, and 1.23 Å, respectively, as shown in Figure S2. The adsorption energy (*E*_ads_) was calculated by subtracting the total energies of the 2D monolayer (*E*_Supercell_/*E*_Alsupercell_) and the nucleobase molecules (*E*_ATCG_) from the total energy of the adsorption system (*E*_(NB/NP/OB/OP)_ /*E*_(AlNB/AlNP/AlOB/AlOP)_).

1 $$E_{ads}=E_{\left(NB/NP/OB/OP\right)}-E_{Supercell}-E_{ATCG}$$

$$E_{ads}=E_{\left(AlNB/AlNP/AlOB/AlOP\right)}-E_{Alsupercell}-E_{ATCG}$$

Figure 3 shows both side and top views of the most stable adsorption structures of Adenine, Thymine, Cytosine, and Guanine nucleobases on pristine BP and Al-doped BP systems. First, Figure 3 a–d illustrates the adsorption configurations of nucleobases on the pristine BP monolayer. Specifically, Adenine and Cytosine are adsorbed via their NH_2_ groups at the P site, labeled as A-BP-NP and C-BP-NP, while Thymine and Guanine are adsorbed via their O atoms at the same site (T-BP-OP and G-BP-OP). The corresponding adsorption energies of Adenine, Thymine, Cytosine, and Guanine nucleobases on the pristine BP are calculated to be −0.63 −0.58, −0.62, and −0.75 eV, respectively. According to the results, no chemical bonding occurs in any of the pristine BP systems, implying that van der Waals interactions mainly drive molecular adsorption.

**Figure 3:**
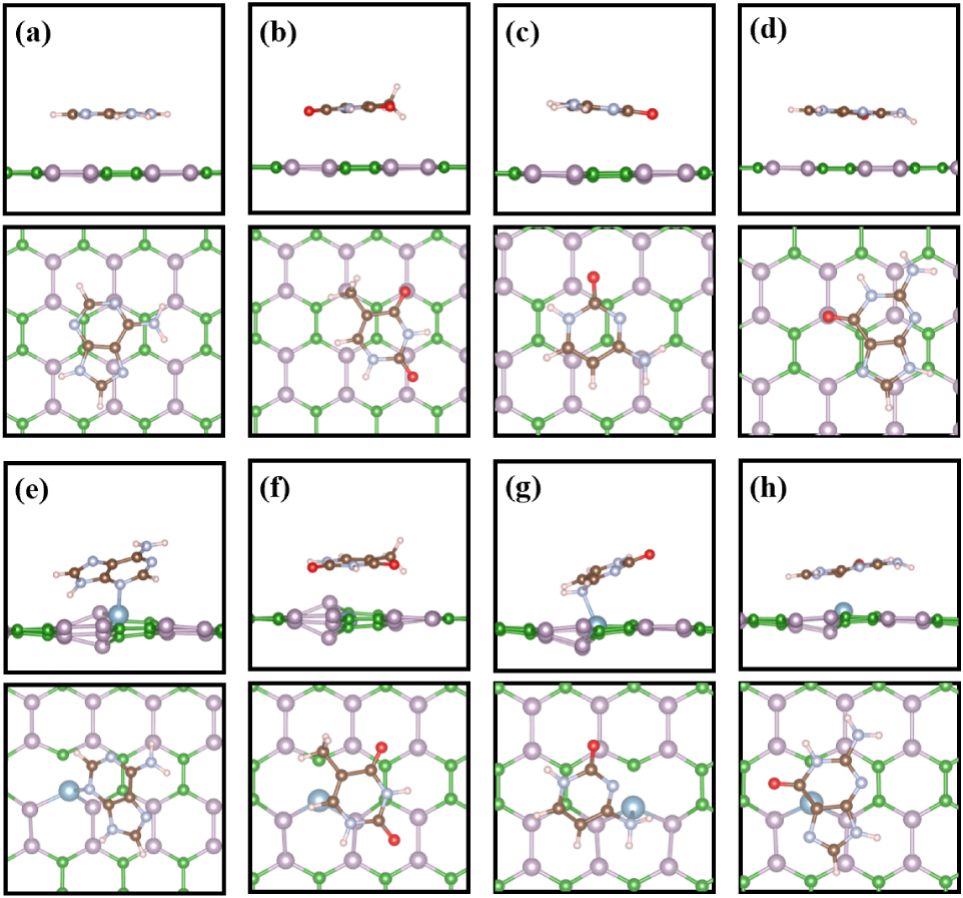
Most stable adsorption configurations of nucleobase molecules on the BP monolayer, presented in both side and top views of (a) A-BP-NP, (b) T-BP-OP, (c) C-BP-NP, and (d) G-BP-OP; (e) A-AlBP-NP, (f) T-AlBP-NB, (g) C-AlBP-NB, and (h) G-AlBP-OP.

On the other hand, Figure 3e–h illustrates the adsorption geometries of the nucleobases on the Al-doped BP monolayer. More precisely, Adenine is adsorbed via its NH_2_ group at the P site (A-AlBP-NP), while Thymine and Cytosine are anchored through their NH_2_ groups at the B site (T-AlBP-NB and C-AlBP-NB). Guanine interacts with the P site via the O atom (G-AlBP-OP). The respective adsorption energies of Adenine, Thymine, Cytosine, and Guanine nucleobases on the Al-doped BP are −2.27 eV, −1.05 eV, −1.80 eV, and −1.46 eV. To elucidate, we found that both A-AlBP-NP and C-AlBP-NB form chemical bonds and tend to adsorb onto the Al atom via their NH_2_ groups rather than through the O atom, indicating a stronger interaction between the NH_2_ group and the Al site. However, Guanine and Thymine could not form the obvious chemical bonds with the Al-doped BP, suggesting a different binding preference, which may be attributed to the attraction between the nucleobases and the P site of the Al-doped BP. These van der Waals-type interactions between the nucleobases and the Al-doped BP are also stronger than those of pristine BP. This result might result from the fact that Al-doped BP can redistribute the electronic states of the monolayer. Then the intermolecular interaction between the nucleobases and the monolayer can be enhanced. Among all of the configurations, A-AlBP-NP exhibits the most negative adsorption energy, indicating that the Adenine molecule possesses the strongest interactions with the Al-doped BP. The adsorption energies of various nucleobase molecules on the pristine and Al-doped BP monolayer are listed in Table 1. Moreover, the calculated gas adsorption heights of the nucleobase molecules on the pristine BP are all larger than 2.50 Å. Still, they are all shorter than 2.34 Å when adsorbing on the Al-doped BP monolayer, as shown in Table 1. These results reveal that the adsorption of the nucleobases on pristine BP is weak. In contrast, the interaction between nucleobase molecules and the Al-doped BP monolayer is strong, exhibiting similar trends in adsorption energies.

**Table 1:** Calculated Adsorption Energy and Adsorption Height of Various Nucleobase Molecules on the Most Stable Sites of the Pristine BP Monolayer and Al-Doped BP Monolayer

	*E*_ads_ (eV)	*h* (Å)
A-BP-NP	–0.63	3.14
T-BP-OP	–0.58	2.81
C-BP-NP	–0.62	2.94
G-BP-OP	–0.75	2.75
A-AlBP-NP	–2.27	2.00
T-AlBP-NB	–1.05	2.34
C-AlBP-NB	–1.80	2.08
G-AlBP-OP	–1.46	2.11

### 2.2 Electron Density Difference (EDD) and Density of States (DOS) Analysis

To analyze the sensing mechanism of nucleobase molecules on the BP and Al-doped BP monolayer, we calculated the electron density difference (EDD) and projected density of states (PDOS) to understand the electronic interactions between nucleobases and 2D materials. For the pristine BP systems, shown in Figure 4a–d, only slight electron accumulation is observed between the molecules and the BP surface, primarily located at the interface, indicating that weak van der Waals interactions dominate the adsorption behavior. By comparison, the Al-doped systems, as shown in Figure 4e–h, exhibit significant charge redistribution occurring at the interface between the nucleobases and the Al-doped BP surface. Particularly, A-AlBP-NP and C-AlBP-NB demonstrate strong electron accumulation and depletion between the NH_2_ groups and the Al-doped monolayer, suggesting that NH_2_ provides electrons to the surface to form N–P and N–B covalent bonds. These results reflect the electronic interactions between molecules and 2D materials, facilitating the formation of the dative chemical bonds.

**Figure 4:**
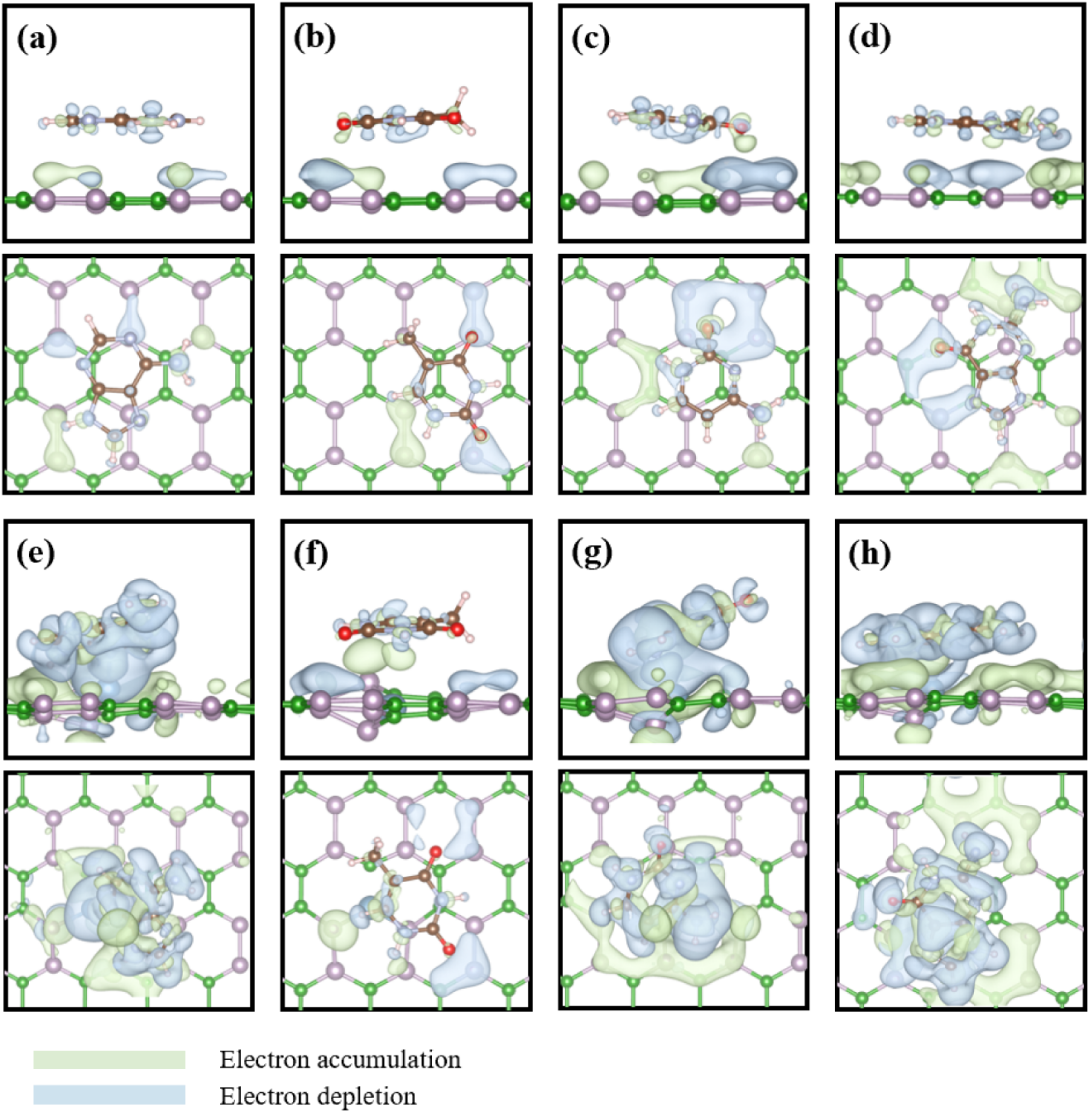
Calculated 3D electron density difference (EDD) plots for the adsorption of nucleobases on the Pristine and Al-doped BP monolayer, presented in both side and top views of (a) A-BP-NP, (b) T-BP-OP, (c) C-BP-NP, and (d) G-BP-OP; (e) A-AlBP-NP, (f) T-AlBP-NB, (g) C-AlBP-NB, and (h) G-AlBP-OP. The green and blue contours represent electron accumulation and depletion, respectively.

Figure 5 shows the density of states (DOS) before adsorption of the pristine BP, Al-doped BP monolayer, and nucleobases. As shown in Figure 5a, the pristine BP exhibits a relatively broad and continuous DOS distribution with noticeable peaks near the valence band maximum (VBM) and conduction band minimum (CBM), characteristic of a typical semiconducting 2D material. Upon aluminum doping, as shown in Figure 5b, the DOS profile undergoes distinct modifications, with the overall shape becoming slightly asymmetric and new localized states emerging near the Fermi level. These changes suggest that Al doping alters the electronic structure, possibly narrowing the band gap and enhancing the material’s electronic sensitivity. In contrast, in Figure 5c–f, the DOS of the nucleobases shows discrete and sharp peaks, which are attributed to the molecular orbitals of these organic compounds.

**Figure 5:**
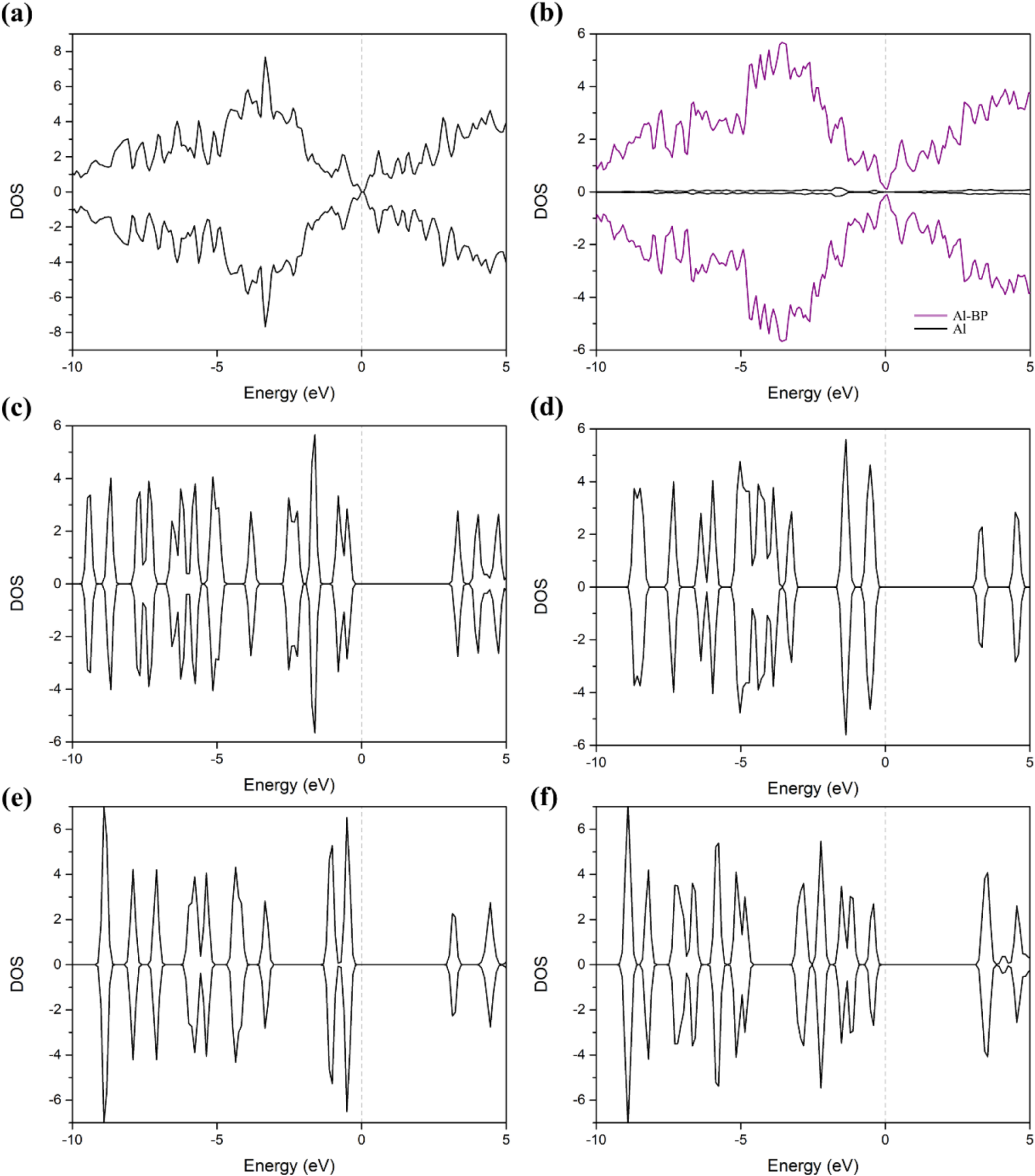
Projected density of states (PDOS) of (a) Pristine BP monolayer, (b) Al-doped BP monolayer, and (c) Adenine, (d) Thymine, (e) Cytosine, and (f) Guanine molecules. The dashed line represents the Fermi energy.

Figure 6 demonstrates the PDOS diagrams after the adsorption of nucleobase molecules on the pristine and Al-doped BP monolayer. For the pristine systems (Figure 6a,c,e,g), weak orbital hybridization is observed between the nucleobase molecules and the BP monolayer. The states from the molecules and the BP surface are mostly separated, with minimal overlap in the energy range from −5 to 0 eV. This indicates that the interaction is mainly physisorption dominated by weak van der Waals forces, which corresponds with the low adsorption energy and minimal charge transfer observed in earlier calculations. In contrast, for the Al-doped systems (Figure 6b,d,f,h), significant changes in the PDOS profiles are observed. The incorporation of Al atoms introduces new states near the Fermi level, particularly between −3 and 0 eV, and these states strongly overlap with the molecular orbitals of the nucleobases. In Figure 6b, which corresponds to the adsorption of Adenine on AlBP at the P site (A-AlBP-NP), pronounced hybridization is evident between the N atoms in the NH_2_ group and the Al-doped BP surface, indicating the formation of covalent N–P bonds. Similarly, in Figure 6f, corresponding to C-AlBP-NB, strong coupling is observed between the NH_2_ group and the B site, suggesting the formation of N–B bonds. Likewise, such orbital hybridization and bond formation are also observed in the other Al-doped systems, further supporting the enhanced interaction between the nucleobases and the doped surface. The black lines in the Al-doped PDOS plots represent the contributions from Al atoms, which appear prominently near the Fermi level and overlap with the molecular states. This overlap highlights the important role of Al in enhancing the electronic coupling and facilitating charge transfer between the base molecules and the BP monolayer. These findings support the results from the electron density difference (EDD) plots and confirm that Al doping significantly increases the electronic sensitivity of the material, enabling a stronger interaction with nucleobases.

**Figure 6:**
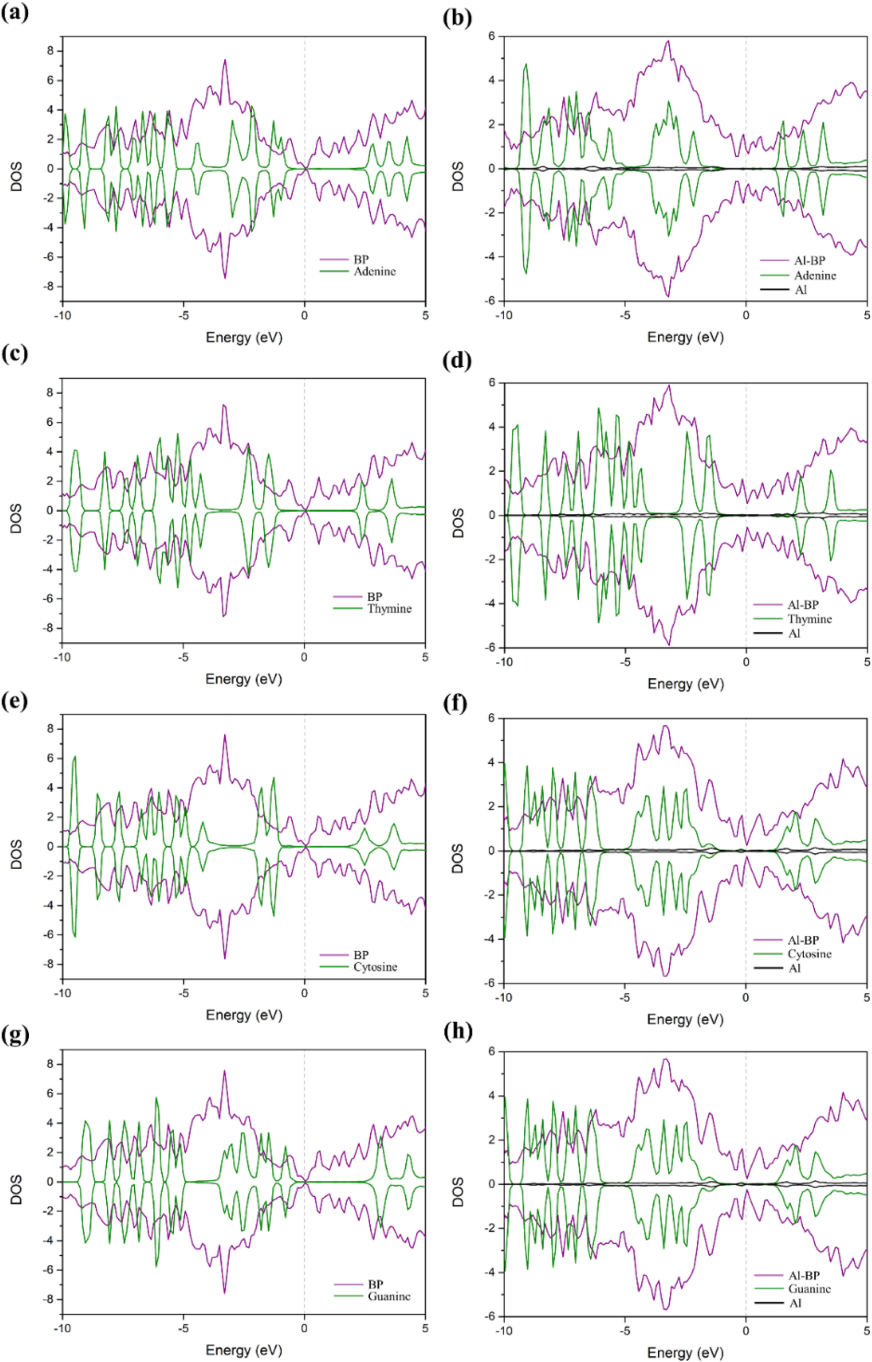
Projected density of states (PDOS) diagrams of (a) A-BP-NP, (b) A-AlBP-NP, (c) T-BP-OP, (d) T-AlBP-NB, (e) C-BP-NP, (f) C-AlBP-NB, (g) G-BP-OP, and (h) G-AlBP-OP. The dashed line represents the Fermi energy.

### 2.3 Electrical Sensitivity

Electrical conductivity plays a vital role in determining the electrical sensitivity. We adopted the Boltzmann transport equation to calculate the electrical conductivity before and after the adsorption of the nucleobases to identify the change in conductivity for the sensitivity. The electrical transport properties were calculated by solving the Boltzmann transport equation with the *BoltzTraP* code.^48^ A constant relaxation time (τ) and a rigid band approximation were employed as standard assumptions in these calculations. Within this approach, the code provides electrical conductivity in the form of σ/τ as a function of both the temperature (*T*) and chemical potential (μ). For the purposes of this work, the conductivity was specifically evaluated at 300 K, representing room temperature conditions.

The calculated electrical conductivity diagrams were depicted in Figure S3. We further calculate the electrical conductivity change by the following formula,

$$\Delta \sigma =\frac{\sigma_{ads}-\sigma_{monolayer}}{\sigma_{monolayer}}$$

Here, σ_ads_ and σ_monolayer_ denote the electrical conductivities of the BP or Al-doped BP monolayer after and before the adsorption of nucleobases, respectively.

In Figure 7a, only a noticeable change in conductivity can be observed between −1.5 eV and −1 eV when Adenine is adsorbed on the pristine BP surface, with the maximum conductivity difference (Δσ) reaching 1.842 at −0.979 eV. In contrast, Figure 7b shows that when Adenine is adsorbed on the Al-doped BP surface, notable conductivity changes occur at −1.888, −1.427, 0.306, and 1.576 eV. In particular, the highest conductivity difference (Δσ) of 7.654 is observed at 1.576 eV. This indicates that Adenine exhibits stronger electrical sensitivity when adsorbed on Al-doped BP than its counterparts on the pristine BP surface, especially in the range from 0 to 2 eV.

**Figure 7:**
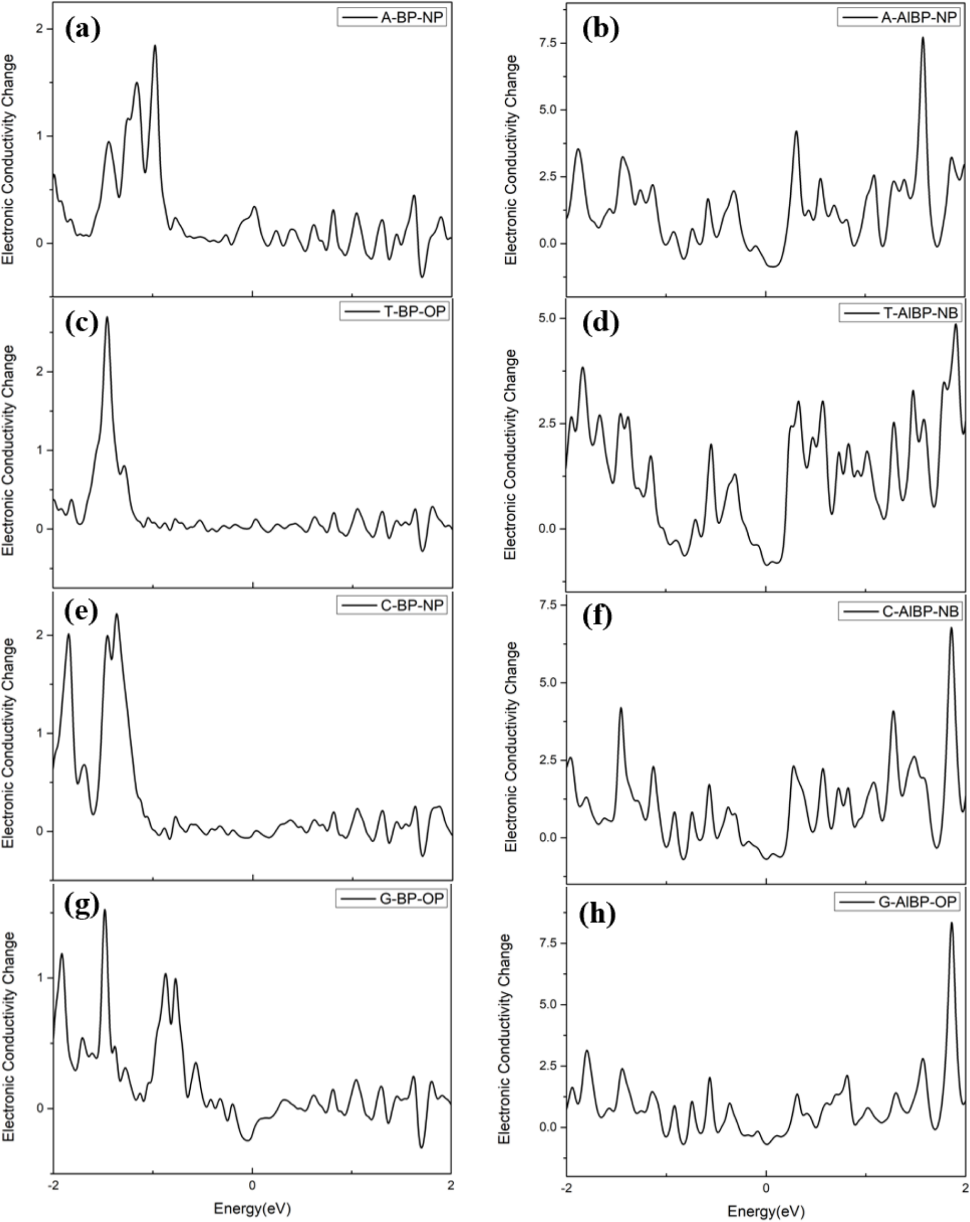
Electrical conductivity changes of (a) A-BP-NP, (b) A-AlBP-NP, (c) T-BP-OP, and (d) T-AlBP-NB; (e) C-BP-NP, (f) C-AlBP-NB, (g) G-BP-OP, and (h) G-AlBP-OP.

Likewise, in Figure 7c, when Thymine is adsorbed on the pristine BP surface, the conductivity change (Δσ) value is calculated as 2.677. From Figure 7d, when Thymine is adsorbed on the Al-doped BP surface, noticeable conductivity changes occur at −1.836 0.334, 0.579, 1.472, and 1.90 eV. The largest conductivity difference (Δσ) of 4.860 appears at 1.90 eV. Besides, when Cytosine is adsorbed on the pristine BP surface and the Al-doped BP surface, as shown in Figure 7e,f, the calculated largest conductivity changes are 2.216 at −1.359 eV and 6.768 at 1.860 eV, respectively. Finally, when Guanine is adsorbed on the pristine BP surface (Figure 7g), it exhibits the smallest conductivity changes in all adsorption cases, ranging from 1.525 at −1.484 eV. However, when Guanine is adsorbed on the Al-doped BP surface, a noticeable conductivity change occurs at −1.864 eV, with a Δσ value reaching 8.304 (Figure 7h). Hence, as summarized in Table 2, our results show that, in comparison with their adsorption on the pristine BP surface, four nucleobase molecules all exhibit excellent electrical sensitivity change when adsorbed on Al-doped BP, in particular for Adenine and Guanine.

**Table 2:** Maximum Conductivity Changes of Various Nucleobases on the Most Stable Sites at the Pristine and Al-Doped BP Surface

	Δσ		Δσ
A-BP-NP	1.842	A-AlBP-NP	7.654
T-BP-OP	2.677	T-AlBP-NB	4.860
C-BP-NP	2.216	C-AlBP-NB	6.768
G-BP-OP	1.512	G-AlBP-OP	8.304

### 2.4 Electronic Band Structure Analysis

The variation in bandgap significantly influences the electrical conductivity of semiconducting chemiresistive gas sensors. This dependence can be expressed through the following relationship between bandgap energy and electrical conductivity:^49–51^

$$\sigma \propto \exp\frac{-E_{g}}{2kT}$$

where σ and *k* are the electrical conductivity and the Boltzmann constant, respectively. *E*_g_ and *T* represent the bandgap values obtained via calculations and at a given temperature, respectively.

As illustrated in Figures S4 and S5, the calculated electronic band structures confirm that the pristine BP monolayer possesses band gaps of 0.34 and 0.56 eV, as determined by the Perdew–Burke–Ernzerhof (PBE) and hybrid Heyd–Scuseria–Ernzerhof (HSE06)^52^ functionals, respectively, which is consistent with previously reported results.^47^ The adsorption of the four nucleobases on pristine BP introduces negligible variations in the bandgap, suggesting that the electrical conductivity remains largely unaffected. In contrast, Al doping results in a modest reduction of the bandgap to 0.25 eV (PBE) and 0.41 eV (HSE06). Further adsorption of Adenine, Thymine, Cytosine, and Guanine on the Al-doped BP monolayer leads to more substantial changes in bandgap values. The PBE calculations yield band gaps of 0.05, 0.10, 0.10, and 0.14 eV, while the HSE06 functional provides slightly larger values of 0.16, 0.20, 0.19, and 0.23 eV, respectively, as listed in Table S1. Among the tested molecules, Adenine produces the most significant reduction in the bandgap, thereby causing the largest alteration in electrical conductivity. This observation is consistent with our conductivity calculations based on the Boltzmann transport equation, confirming that the Al-doped BP monolayer exhibits the highest sensitivity toward Adenine adsorption. These results highlight the pivotal role of Al doping in modulating the electronic response of BP and underline its potential in selective molecular sensing applications.

### 2.5 Electron Localization Function (ELF) Analysis

We have calculated the electron localization function (ELF) plots to describe the bonding characteristics of the nucleobase’s adsorption. According to the ELF plots, as shown in Figure S6, all of the nucleobases show no electron density between the molecules and the pristine BP monolayer. This indicates that no actual chemical bonds are formed between the molecules and the pristine BP monolayer. On the other hand, both Adenine and Cytosine molecules form actual chemical bonds with the Al atom on the Al-doped BP monolayer. The Guanine molecule also exhibits a strong interaction with the Al atom in the Al-doped BP monolayer, although it does not form a chemical bond. Only Thymine shows no electron density to the Al-doped BP monolayer, indicating a weak interaction. The results demonstrate that the stronger interaction, as indicated by the electron localization function plots, causes a larger adsorption energy of the nucleobase molecules.

### 2.6 Optical Sensitivity Property

Figures 8 and 9 illustrate the optical sensitivity of the systems before and after aluminum doping. As shown in Figure 8, which displays the undoped configurations, the absorption peaks for the nucleobase-adsorbed BP monolayers are as follows: A-BP-NP exhibits a peak at 441.03 nm, T-BP-OP at 443.18 nm, C-BP-NP at 442.56 nm, and G-BP-OP at 446.74 nm. These values are only slightly shifted from the pristine BP monolayer, which has an intrinsic absorption peak at 440.28 nm. The minimal changes in the absorption peak positions suggest that the undoped BP monolayer has weak optical sensitivity to the adsorption of nucleobases. In other words, despite molecular adsorption, the optical response of the material remains relatively unchanged, limiting its applicability in optical sensing.

**Figure 8:**
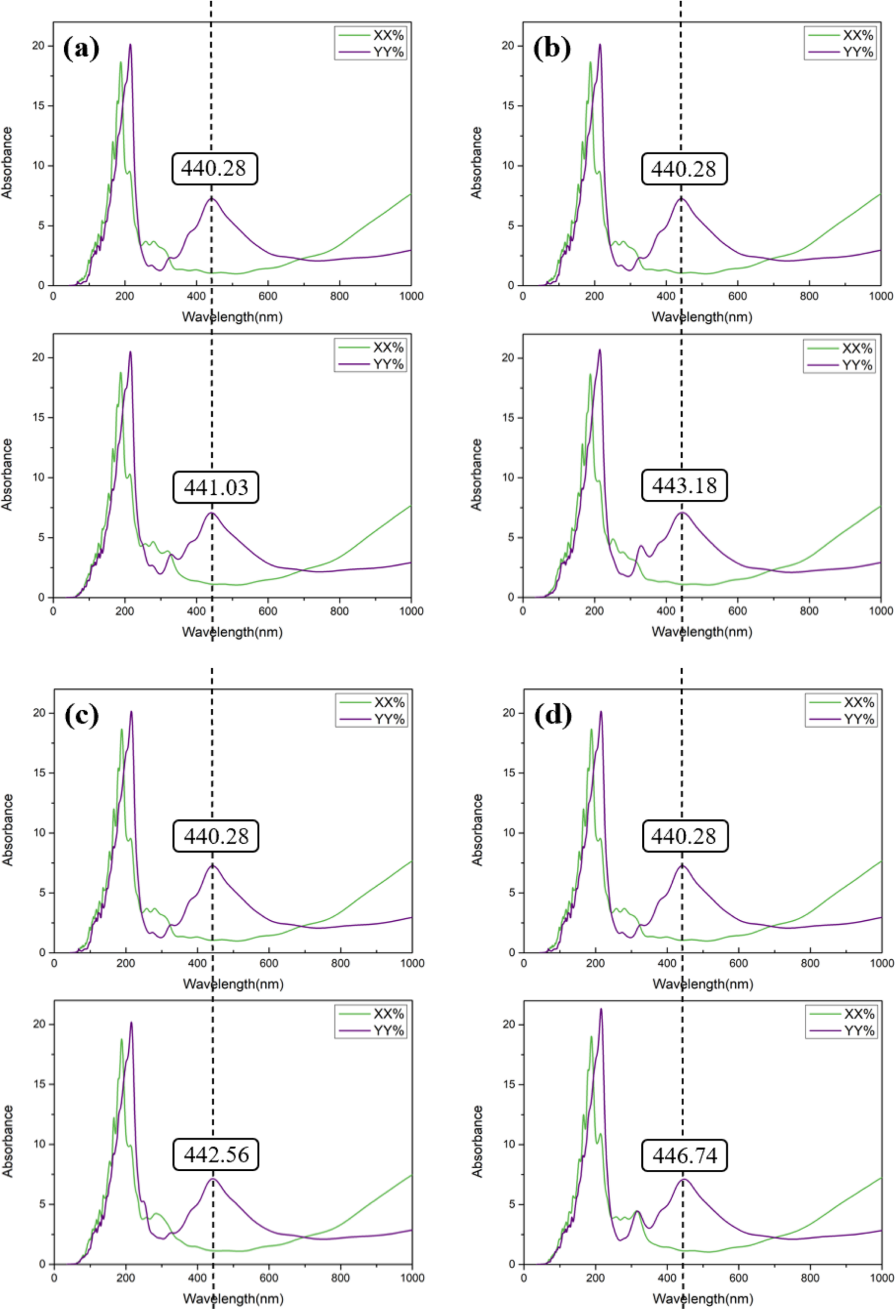
Simulated optical absorption spectrum of (a) A-BP-NP, (b) T-BP-OP, (c) C-BP-NP, and (d) G-BP-OP in comparison to the pristine BP monolayer.

**Figure 9:**
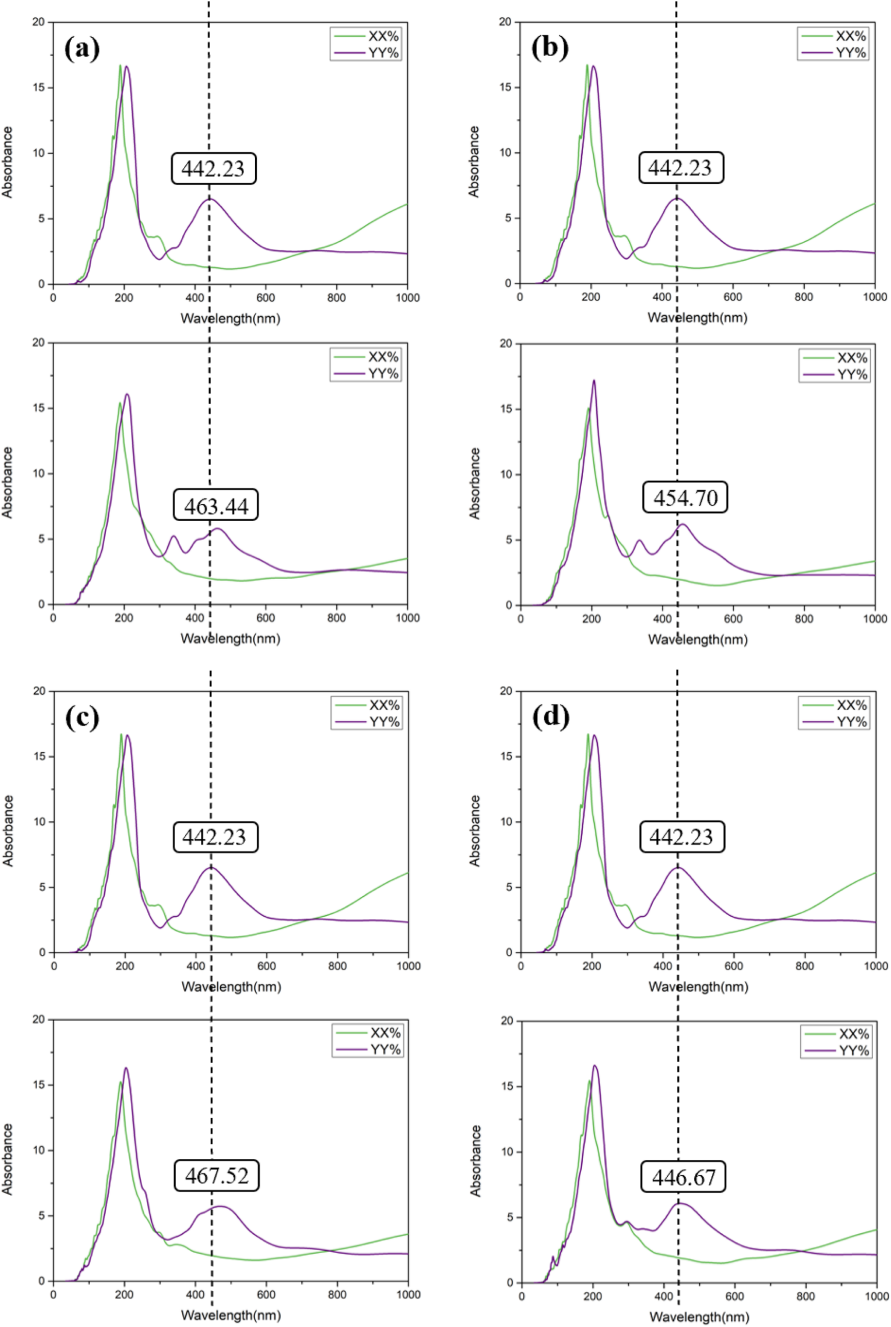
Simulated optical absorption spectra of (a) A-AlBP-NP, (b) T-AlBP-NB, (c) C-AlBP-NB, and (d) G-AlBP-OP in comparison with the Al-doped BP monolayer.

In contrast, Figure 9 presents the optical behavior of the Al-doped configurations. Upon doping, the absorption peaks shift more noticeably: A-AlBP-NP shows a significant redshift to 463.44 nm, T-AlBP-NB to 454.70 nm, C-AlBP-NB to 467.52 nm, and G-AlBP-OP to 446.67 nm. Compared to the intrinsic peak of 442.23 nm for the aluminum-doped BP monolayer, these redshifts indicate that the interaction with nucleobases leads to a more substantial change in the optical response. This demonstrates that aluminum doping not only modifies the electronic structure of the BP monolayer but also significantly enhances its optical sensitivity.

Among all configurations, A-AlBP-NP exhibits the largest shift in its absorption peak, suggesting that it has the highest optical sensitivity among the systems studied. These findings confirm that Al-doped BP is a more promising platform for optical sensing applications than its undoped counterpart. A detailed summary of the absorption peaks corresponding to the optimal adsorption configurations is presented in Table 3.

**Table 3:** Calculated Absorption Wavelengths of Various Nucleobases on the Most Stable Sites at the Pristine and Al-Doped BP Surface

	Wavelength (nm)		Wavelength (nm)
BP	440.28	Al-BP	442.23
A-BP-NP	441.03	A-AlBP-NP	463.44
T-BP-OP	443.18	T-AlBP-NB	454.70
C-BP-NP	442.56	C-AlBP-NB	467.52
G-BP-OP	446.74	G-AlBP-OP	446.67

### 2.7 Selectivity Analysis

The variation in monolayer work function (Φ) resulting from molecular adsorption serves as the basis for electronic molecular detection, wherein greater work function changes correspond to enhanced detectability. The work function values were obtained directly from the VASP output using the standard electrostatic potential method. During the self-consistent calculations, the local electrostatic potential was enabled (LVTOT = .TRUE.; and LVHAR = .TRUE.), generating the LOCPOT file containing the potential distribution across the supercell. From this file, the planar-averaged potential along the surface normal (*z*-axis) was extracted. In the vacuum region between periodic slabs, the potential reaches a plateau, which defines the vacuum energy level (*E*_vac_). The Fermi energy (*E*_f_) was taken from the OUTCAR file after convergence of the electronic structure. Then the work function was evaluated as,

$$\Phi =E_{vac}-E_{f}$$

A larger change in the work function before and after molecular adsorption indicates a stronger electron transfer between the adsorbate and the surface, implying a higher sensitivity and easier detection of the target molecules.

Figure 10a,b presents the calculated work functions of pristine BP and Al-doped BP systems, before and after the adsorption of nucleobases. As shown in Figure 10a, in the pristine BP systems, the work functions of T-BP-OP and C-BP-NP remain unchanged at 4.727 eV, identical to those of pristine BP. This suggests that these adsorption configurations exert minimal influence on the surface electron migration. In contrast, A-BP-NP and G-BP-OP exhibit slightly increased work functions of 4.741 and 4.786 eV, respectively, indicating a moderate suppression of electron mobility due to adsorption. Figure 10b clearly demonstrates that Al doping significantly reduces the work function of pristine BP to 4.632 eV, implying that the introduction of Al atoms effectively lowers the energy barrier for electron emission, thereby potentially enhancing electron transport. Following adsorption, the work functions of A-AlBP-NP and G-AlBP-OP slightly increase to 4.736 and 4.741 eV, respectively, suggesting that electron migration is still suppressed by adsorption. Notably, T-AlBP-NB and C-AlBP-NB exhibit further increased work functions of 4.753 and 4.790 eV, respectively. These results imply a synergistic effect between adsorption and Al doping, which enhances the surface’s electron-binding capability.

**Figure 10:**
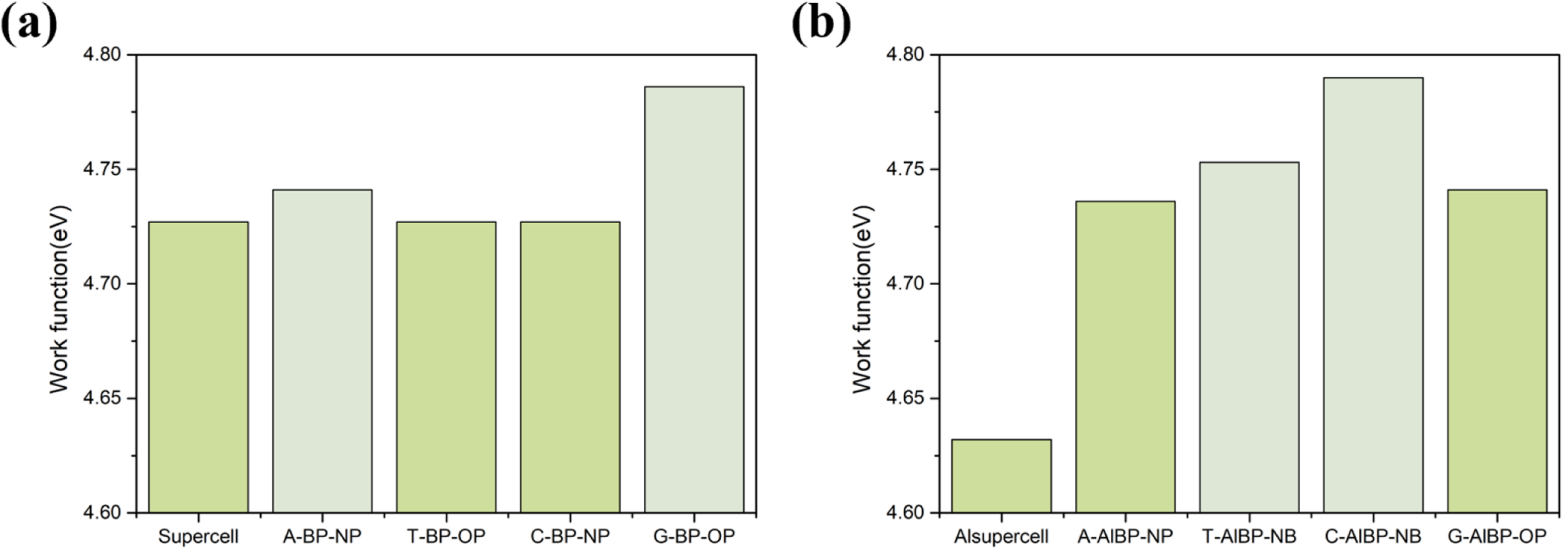
Calculated work function values of the adsorption for the nucleobases on (a) the pristine BP monolayer and (b) the Al-doped BP monolayer.

Figure 11 presents a comparative analysis of the selectivity of the studied systems. Selectivity refers to the material’s ability to distinguish between different target molecules, which is a critical parameter in sensor design. As shown in Figure 11a, under pristine conditions, Guanine exhibits the highest selectivity among all of the nucleobases, suggesting that pristine BP has a stronger discriminatory response toward Guanine. This implies that Guanine induces a more significant electronic change in the system compared to other molecules, making it easier for the material to differentiate itself. In contrast, Figure 11b illustrates the selectivity under aluminum-doped conditions. In this case, Adenine demonstrates the highest selectivity, indicating a notable shift in the interaction mechanism after doping. The enhanced electronic response induced by Adenine adsorption on the Al-doped BP surface leads to improved selectivity. This change suggests that aluminum doping alters the surface electronic environment of BP, enabling the material to respond to and distinguish Adenine from the other bases more effectively. This comparison highlights the influence of doping on the selectivity characteristics of the material and underscores the tunability of Al-doped BP in tailoring the sensor performance for specific target molecules.

**Figure 11:**
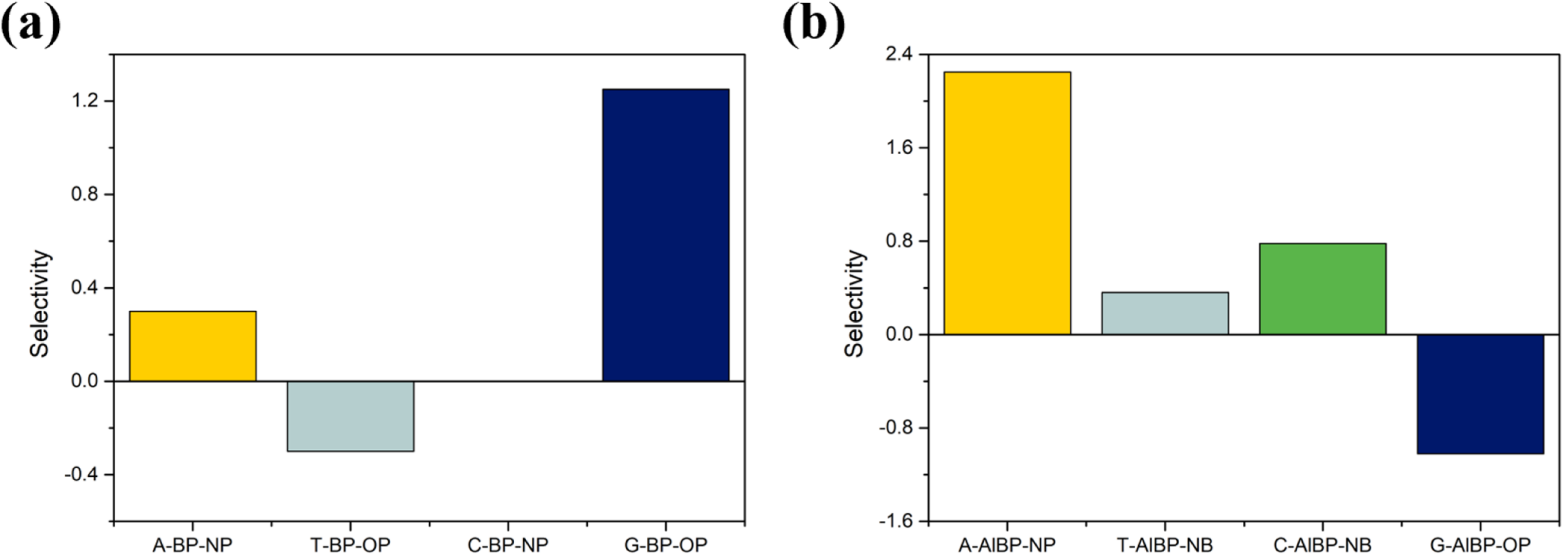
Calculated selectivity based on the work function of the adsorption for the nucleobases on (a) the pristine BP monolayer and (b) the Al-doped BP monolayer.

We further compared our adsorption energy values of the nucleobase molecules with the previously calculated theoretical results from the literature, as shown in Table 4. The adsorption energy of Adenine on the Al-doped BP monolayer demonstrates the strongest binding strength compared to previous studies. In summary, the Al-doped BP monolayer not only enhances molecular adsorption but also exhibits excellent electrical and optical sensitivity to the nucleobase molecules, as summarized in Table 5. Thus, the aluminum-doped boron phosphide 2D materials emerge as the most promising biosensor for detecting Adenine.

**Table 4:** Comparison of Adsorption Energies of the Nucleobases on Various Gas Sensors

	*E*_ads_ (eV)			
Sensor	Adenine	Thymine	Cytosine	Guanine
Silicene^17^	–1.07	–0.73	–1.19	–1.35
Germanene^17^	–0.87	–0.83	–1.03	–1.30
Au-decorated silicene^17^	–1.35	–1.29	–1.46	–1.84
Li-decorated silicene^17^	–1.56	–1.61	–2.09	–2.30
Au-decorated germanene^17^	–1.50	–1.33	–1.52	–1.75
Li-decorated germanene^17^	–1.58	–1.67	–2.06	–2.28
Pristine silicon nanowire^16^	–1.74	–1.64	–1.72	–2.12
Cu-functionalized SiNW^16^	–1.17	–0.68	–1.55	–1.32
Ag-functionalized SiNW^16^	–1.04	–0.43	–1.25	–1.05
Au-functionalized SiNW^16^	–1.00	–0.78	–1.24	–1.09
Delta Tellurene^21^	–0.67	–0.64	–0.68	–0.83
Pristine boron phosphide	–0.63	–0.58	–0.62	–0.75
Al-doped boron phosphide	–2.27	–1.05	–1.80	–1.46

**Table 5:** Comparison of the Optical and Electrical Sensitivity of Various Nucleobases on the Most Stable Sites at the Pristine and Al-Doped BP Surface

	Optic/Electrical		Optic/Electrical
A-BP-NP	Poor/Poor	A-AlBP-NP	Good/Good
T-BP-OP	Poor/Poor	T-AlBP-NB	Good/Medium
C-BP-NP	Poor/Poor	C-AlBP-NB	Medium/Medium
G-BP-OP	Poor/Poor	G-AlBP-OP	Poor/Good

## 3 Conclusions

In this study, density functional theory (DFT) calculations were employed to investigate the adsorption behavior of nucleobase molecules on both pristine boron phosphide (BP) and aluminum-doped boron phosphide (Al-doped BP) two-dimensional materials. First, we calculated and compared the adsorption energies of each nucleobase molecule on both pristine BP and Al-doped BP monolayers. We observed that the Al-doped BP monolayer can enhance the adsorption strength toward nucleobase molecules. Besides, both Adenine and Cytosine were found to produce an Al–N dative chemical bond between the molecules and the Al-doped BP monolayer. Among these, the adsorption of Adenine on the Al-doped BP (A-AlBP-NP configuration) exhibits the highest adsorption energy of −2.27 eV, indicating it has the strongest chemical interaction with the Al-doped BP monolayer.

Subsequently, we evaluated the electrical sensitivity of each adsorption configuration. Based on the change in conductivity (Δσ) induced by molecular adsorption, Adenine and Guanine exhibit large conductivity variations of 7.654 and 8.304, respectively, significantly higher than those of the other nucleobases, suggesting superior electrical sensitivity. Cytosine also demonstrated good electrical sensitivity with Δσ values of 6.768. In contrast, Thymine showed a relatively low sensitivity. Finally, the optical absorption characteristics were analyzed to assess the optical sensitivity of the systems. According to the optical absorption spectra, all Al-doped configurations exhibit more pronounced shifts in absorption peaks compared with their undoped counterparts. Consistent with its strong adsorption performance, the adsorption of Adenine on the Al-doped BP also shows the largest redshift among all configurations, indicating the highest level of optical sensitivity.

In summary, aluminum-doped boron phosphide 2D materials not only enhance molecular adsorption but also exhibit excellent electrical and optical sensitivity. By integrating the results from both optical and electrical analyses, the aluminum-doped boron phosphide 2D materials emerge as the most promising biosensor for detecting Adenine due to their outstanding selectivity and sensing performance. Recently, the machine learning interatomic potentials (MLIPs)^53^ offered an excellent approach for facilitating calculations at the large-scale level, combining the accuracy of DFT with the efficiency of MD. Although we did not include it in the present work, we acknowledge that MLIPs could be valuable for future extensions of this work. The MLIPs may be very useful for investigating the coadsorption of multiple nucleobase molecules, allowing one to determine which nucleobase molecule is more likely to be adsorbed on the surface based on large-scale calculations in the future.

## 4 Computational Details

Density functional theory (DFT) calculations were carried out using the Vienna *ab initio* simulation package (VASP).^54–57^ The exchange–correlation effects were treated within the generalized gradient approximation (GGA) using the Perdew–Burke–Ernzerhof (PBE) functional.^58^ Core–valence interactions were represented by projector augmented wave (PAW) pseudopotentials,^59,60^ and the plane-wave basis set was truncated at a kinetic energy cutoff of 400 eV. Gas adsorption was investigated using a (3 × 3 × 1) Monkhorst–Pack k-point mesh.^61^ For the self-consistent electronic loop, the Blocked–Davidson scheme was applied with an energy convergence threshold of 1 × 10^–5^ eV, while structural relaxations were performed using the Conjugate Gradient algorithm until all atomic forces were less than 2 × 10^–2^ eV/Å. Dispersion interactions between adsorbates and the monolayer were accounted for using the Grimme DFT-D3 correction method.^62,63^ The convergence tests for various cutoff energies for the adsorption energy calculation of the nucleobase were listed in Table S2, where the adsorption energies have the same trends by using a larger kinetic energy cutoff (500 and 600 eV) as the results by using a 400 eV cutoff energy. Thus, the cutoff energy of 400 eV is still reasonable in this study. Spin-polarization calculations and dipole correction calculations were also involved in the present work. For the spin-polarization calculations, the results show that both pristine BP and Al-doped BP systems, before and after the adsorption of nitrogenous base molecules, are nonmagnetic, indicating that spin-polarization does not affect the adsorption energy (Figures 5 and 6). Besides, when considering the dipole corrections, we found that there are negligible changes (<0.02 eV adsorption energy) for both pristine BP and Al-doped BP systems before and after the adsorption of the nitrogenous base molecules. Thus, both spin-polarization and dipole correction calculations show limited effects on the adsorption energies of the nucleobases, as listed in Table S3.

The optical absorption characteristics for the sensing nucleobase molecules on pristine BP and Al-doped BP monolayers were investigated through calculations based on the frequency-dependent dielectric function, defined as ε­(ω) = ε_1_(ω) + iε_2_(ω).^64,65^ The imaginary part, ε_2_(ω), was determined by evaluating the momentum matrix elements between the occupied and unoccupied electronic states. Subsequently, the real part, ε_1_(ω), was obtained by applying it to the Kramers–Kronig relation. Based on these components, the optical absorption spectrum was derived using the absorption coefficient, α­(ω), which is expressed by the following equation:

$$\alpha \left(\omega \right)=\sqrt{2}\omega {\left[\sqrt{\varepsilon_{1}{\left(\omega \right)}^{2}+\varepsilon_{2}{\left(\omega \right)}^{2}}-\varepsilon_{1}\left(\omega \right)\right]}^{1/2}$$

Postprocessing of the band structure and optical data was performed conveniently with the help of the VASPKIT utility.^66^

## Supplementary Material



## Notes

Calculated energy band gap by PBE and HSE06 functionals (in eV) before and after the adsorption of the nucleobase molecules on both pristine BP and Al-doped BP monolayer (Table S1); convergence tests for various cutoff energy values for the adsorption energy of the nucleobase molecules on both pristine BP and Al-doped BP monolayer (Table S2); calculated adsorption energy of various nucleobase molecules on the pristine BP monolayer and Al-doped BP monolayer with and without dipole corrections (Table S3); top views of unit-cell and supercell of the pristine BP monolayer (Figure S1); calculated bond lengths of the nucleobase structures of Adenine, Thymine, Cytosine, and Guanine (Figure S2); electronic conductivity of various nucleobase molecules on both pristine BP and Al-doped BP monolayer before and after adsorption (Figure S3); calculated electronic band structures via PBE and HSE06 functionals of various nucleobase molecules on both pristine BP and Al-doped BP monolayer before and after adsorption, respectively (Figures S4 and S5); calculated electron localization function (ELF) diagrams of various nucleobase molecules on both pristine BP and Al-doped BP monolayer before and after adsorption (Figure S6) (PDF)
