# Supplementary material for: Electronic and Optical Sensitivity of Aluminum-Doped Boron Phosphide Monolayers for DNA Base Detection: A First-Principles Study
Source: ACS Omega. 2025 Dec 2;10(49):60627–40. doi: 10.1021/acsomega.5c08476 (PMC12713493; doi:10.1021/acsomega.5c08476)
Supplement: Supplementary file 1 [file ao5c08476_si_001.pdf]

Supporting Information:

Electronic and Optical Sensitivity of Aluminum-Doped Boron Phosphide Monolayers for DNA Base Detection: A First-Principles Study

Sin Ye and Chen-Hao Yeh*

Department of Materials Science and Engineering, Feng Chia University, No. 100, Wenhwa Rd., Seatwen, Taichung, 40724, Taiwan

*Corresponding author. E-mail: chenhyeh@fcu.edu.tw (C.-H. Yeh)

Table S1. Calculated energy band gap by PBE and HSE06 functionals (in eV) before and after the adsorption of the nucleobase molecules on both pristine BP and Al-doped BP monolayer.

	E_g^{PBE} (eV)	E_g^{HSE06} (eV)
BP	0.34	0.56
A-BP-NP	0.36	0.57
T-BP-OP	0.35	0.57
C-BP-NP	0.34	0.56
G-BP-OP	0.33	0.55
Al-BP	0.25	0.41
A-AlBP-NP	0.05	0.16
T-AlBP-NB	0.10	0.20
C-AlBP-NB	0.10	0.19
G-AlBP-OP	0.14	0.23

Table S2. Convergence tests for various cutoff energy values for the adsorption energy of the nucleobase molecules on both pristine BP and Al-doped BP monolayer.

	A-BP-NP	T-BP-OP	C-BP-NP	G-BP-OP
	E_{ads} (eV)	E_{ads} (eV)	E_{ads} (eV)	E_{ads} (eV)
400 eV	-0.63	-0.58	-0.62	-0.75
500 eV	-0.62	-0.58	-0.62	-0.75
600 eV	-0.62	-0.58	-0.61	-0.74
	A-AlBP-NP	T-AlBP-NB	C-AlBP-NB	G-AlBP-OP
	E_{ads} (eV)	E_{ads} (eV)	E_{ads} (eV)	E_{ads} (eV)
400 eV	-2.27	-1.05	-1.80	-1.46
500 eV	-2.28	-1.04	-1.79	-1.47
600 eV	-2.28	-1.04	-1.79	-1.46

Table S3. Calculated adsorption energy of various nucleobase molecules on the pristine BP monolayer and Al-doped BP monolayer with and without dipole corrections.

	E_{ads}(eV) without dipole corrections	E_{ads}(eV) with dipole corrections
A-BP-NP	-0.63	-0.63
T-BP-OP	-0.58	-0.58
C-BP-NP	-0.62	-0.62
G-BP-OP	-0.75	-0.76
A-AlBP-NP	-2.27	-2.26
T-AlBP-NB	-1.05	-1.04
C-AlBP-NB	-1.80	-1.79
G-AlBP-OP	-1.46	-1.46

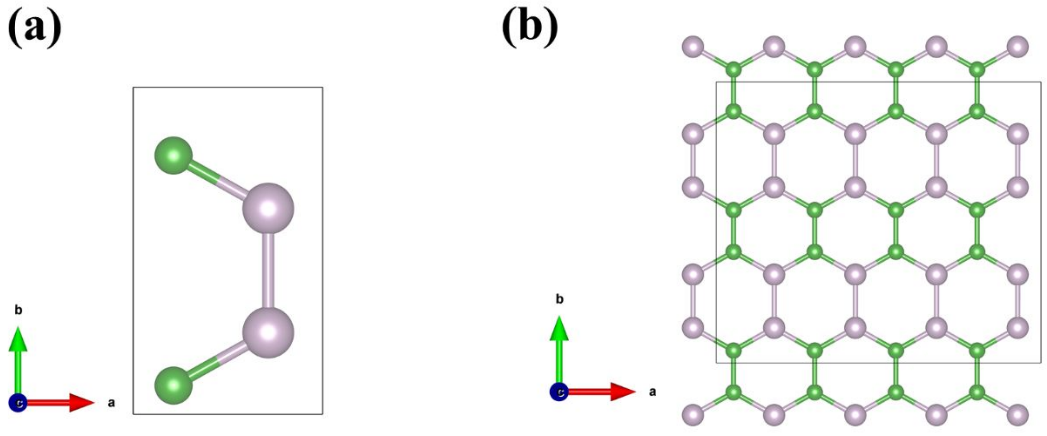

Figure S1. Top views of (a) unit-cell and (b) supercell of the pristine BP monolayer.

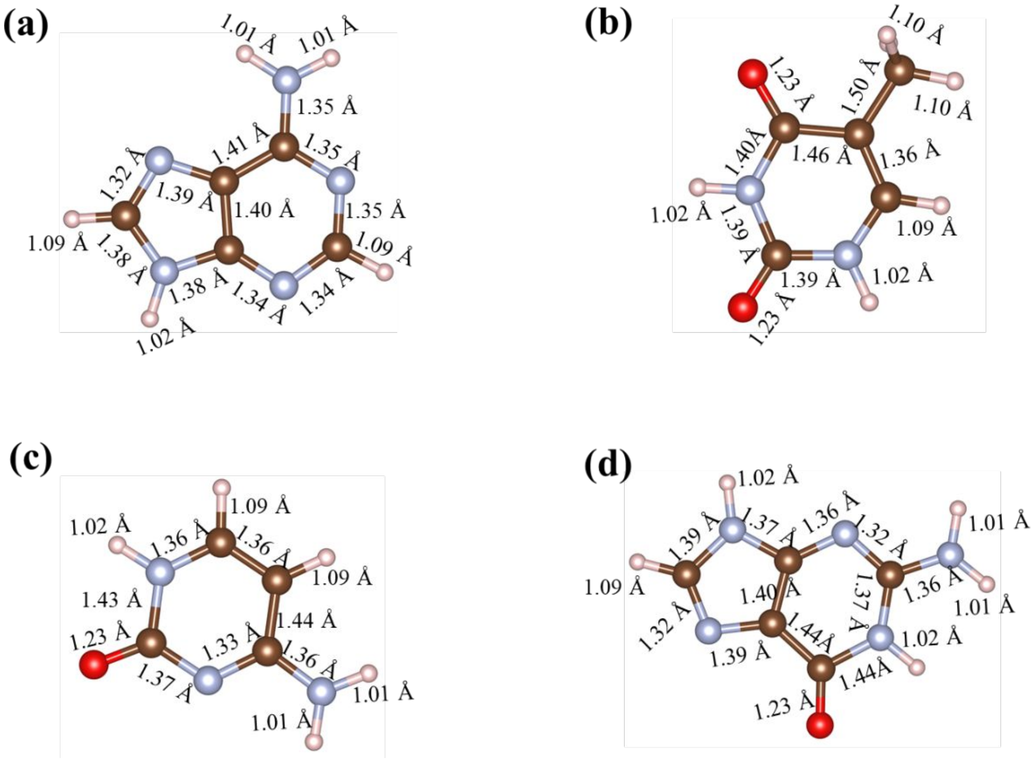

Figure S2. Calculated bond lengths of the nucleobase structures: (a) Adenine, (b) Thymine, (c) Cytosine, and (d) Guanine.

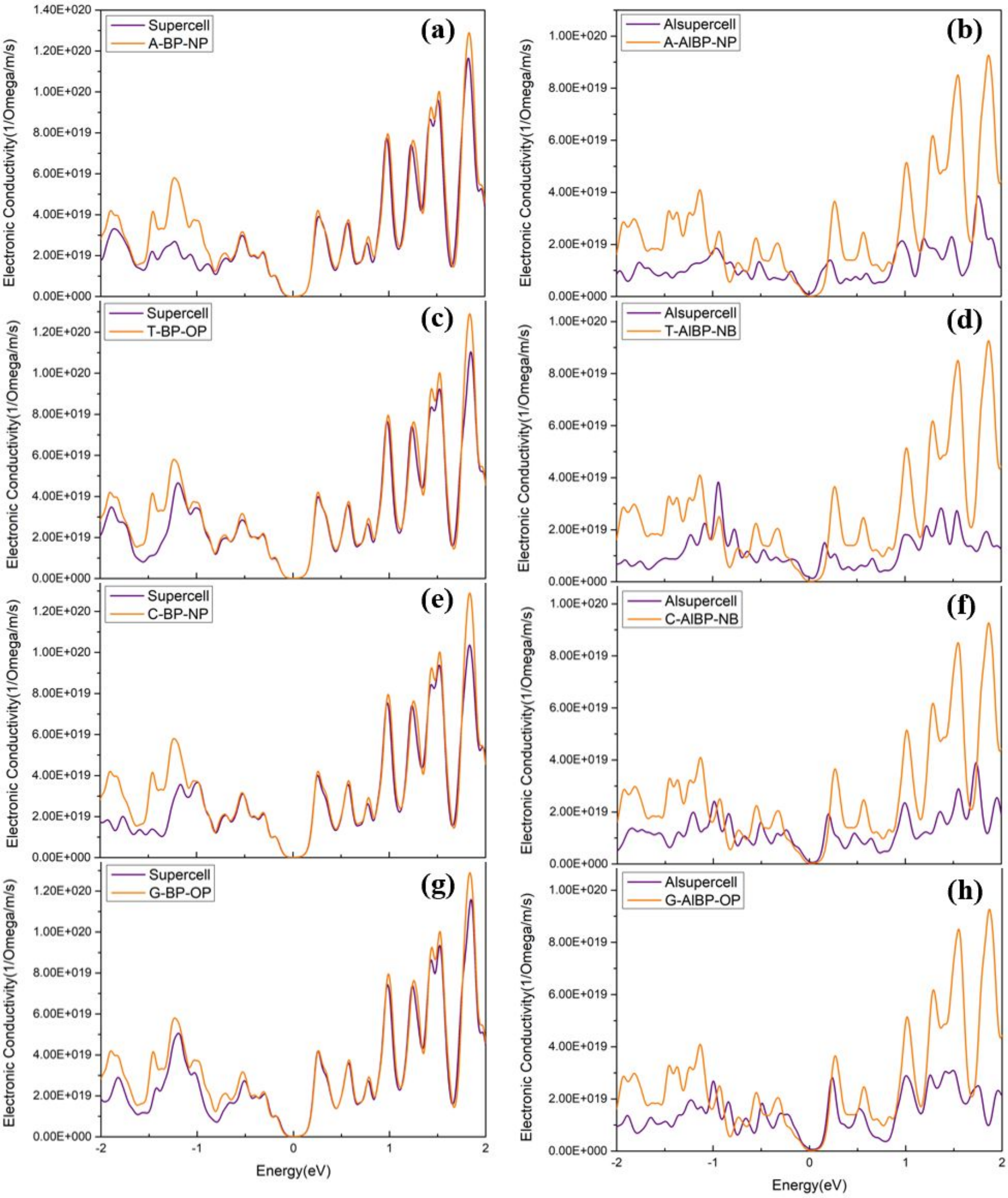

Figure S3. Electronic conductivity of (a) A-BP-NP, (b) A-AlBP-NP, (c) T-BP-OP, and (d) T-AlBP-NB; (e) C-BP-NP, (f) C-AlBP-NB, (g) G-BP-OP and (h) G-AlBP-OP.

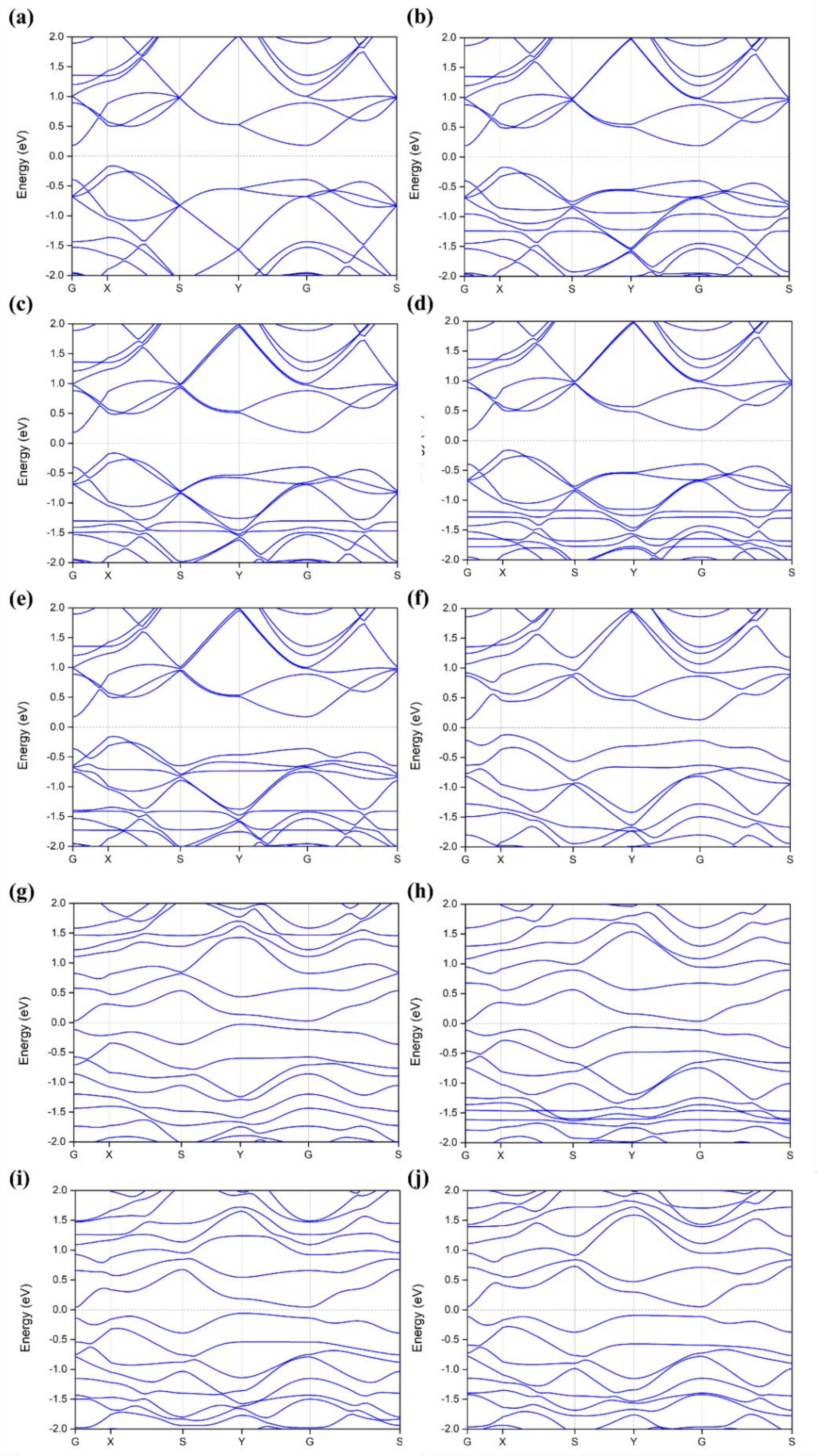

Figure S4. Calculated electronic band structures via PBE functional of (a) pristine BP, (b) A-BP-NP, (c) T-BP-OP, (d) C-BP-NP, and (e) G-BP-OP; and (f) Al-doped BP, (g) A-AlBP-NP, (h) T-AlBP-NB, (i) C-AlBP-NB, and (j) G-AlBP-OP.

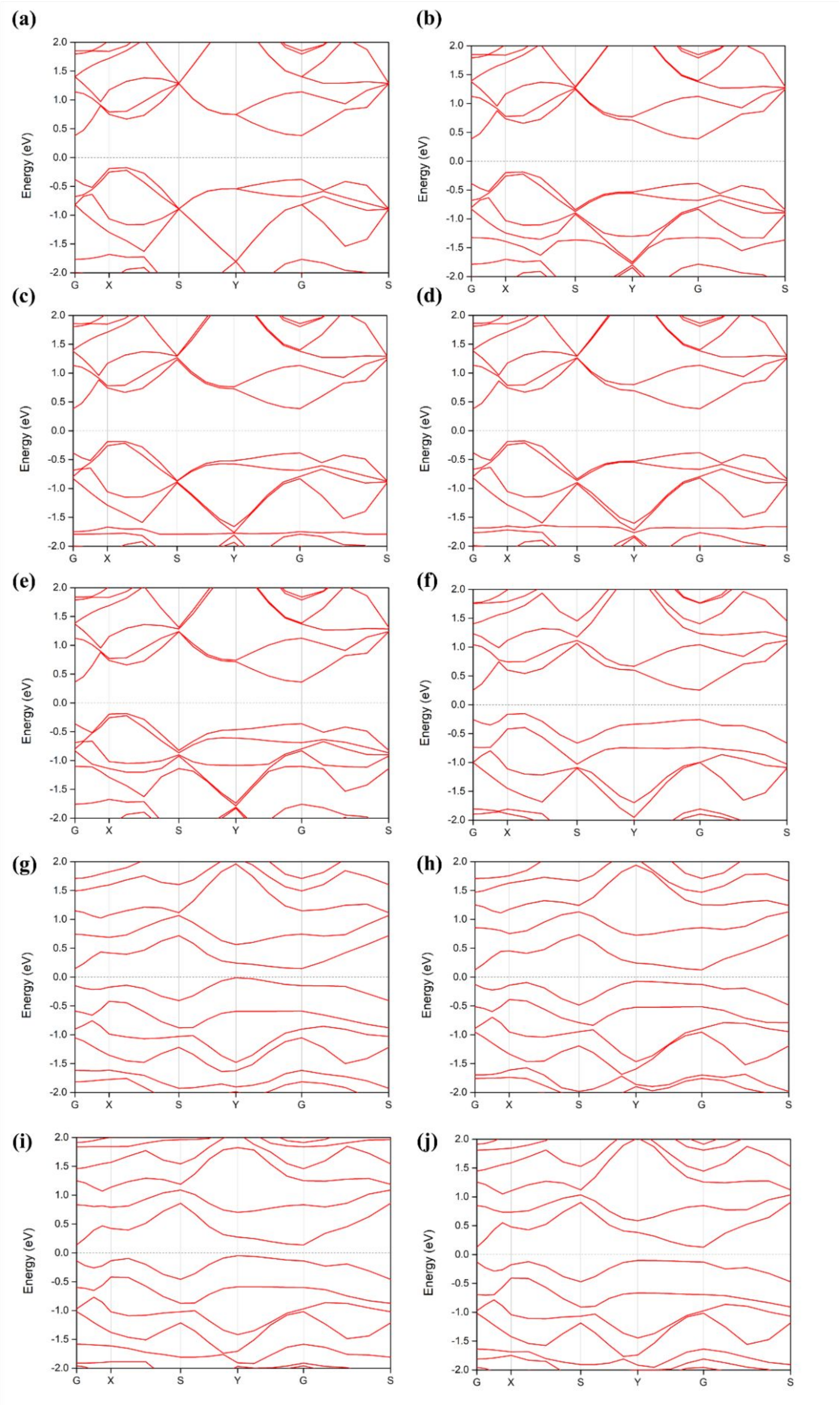

Figure S5. Calculated electronic band structures via HSE06 functional of (a) pristine BP, (b) A-BP-NP, (c) T-BP-OP, (d) C-BP-NP, and (e) G-BP-OP; and (f) Al-doped BP, (g) A-AlBP-NP, (h) T-AlBP-NB, (i) C-AlBP-NB, and (j) G-AlBP-OP.

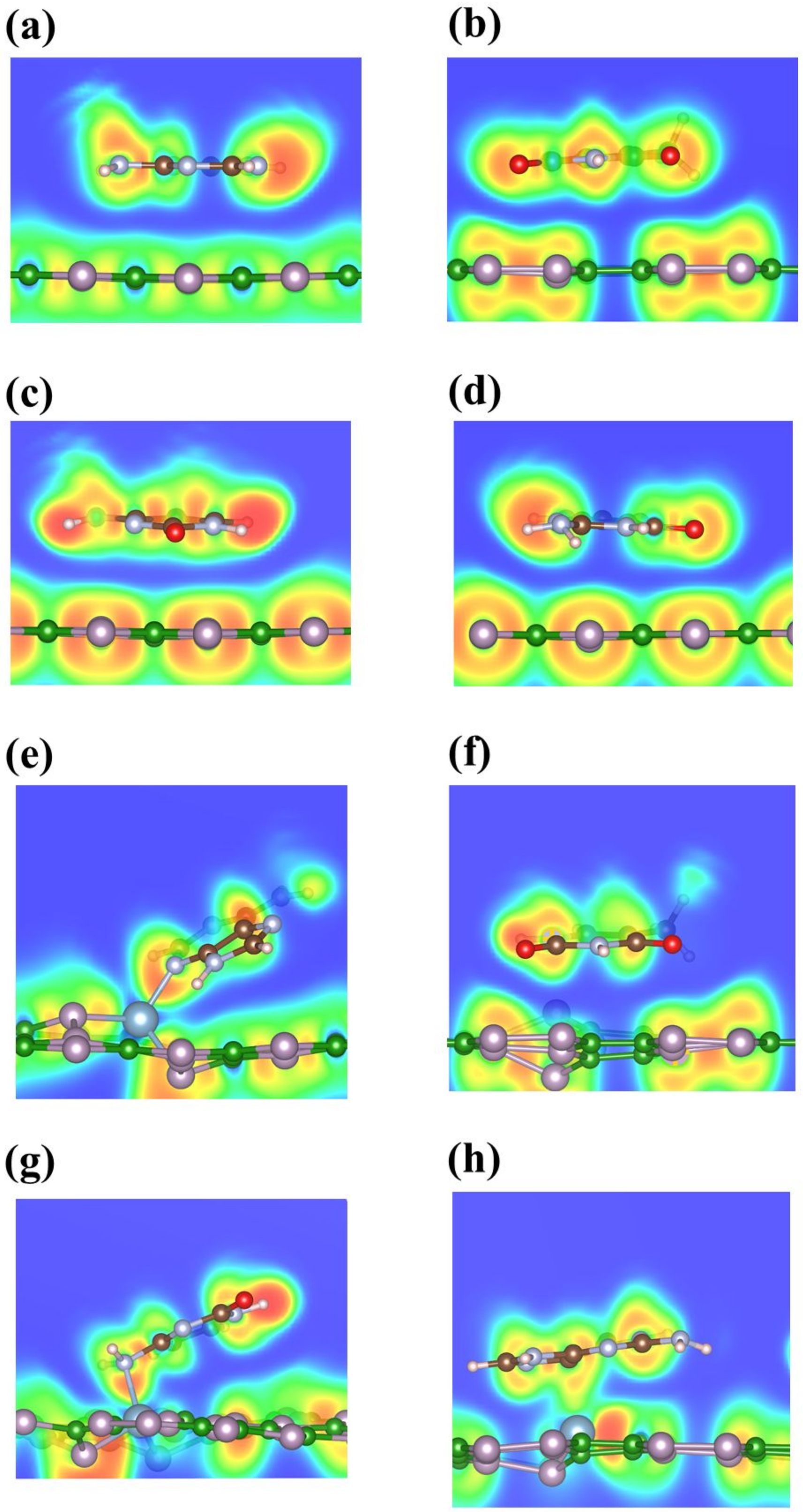

Figure S6. Calculated electron localization function (ELF) diagrams of (a) A-BP-NP, (b) T-BP-OP, (c) C-BP-NP, (d) G-BP-OP, (e) A-AlBP-NP, (f) T-AlBP-NB, (g) C-AlBP-NB, and (h) G-AlBP-OP.
